# Incidence and risk factors for hypertension among HIV patients in rural Tanzania – A prospective cohort study

**DOI:** 10.1371/journal.pone.0172089

**Published:** 2017-03-08

**Authors:** Eduardo Rodríguez-Arbolí, Kim Mwamelo, Aneth Vedastus Kalinjuma, Hansjakob Furrer, Christoph Hatz, Marcel Tanner, Manuel Battegay, Emilio Letang, on behalf of the KIULARCO Study Group

**Affiliations:** 1 Virgen del Rocío University Hospital, Seville, Spain; 2 Ifakara Health Institute, Ifakara, Tanzania; 3 Department of Infectious Diseases, Bern University Hospital and University of Bern, Bern, Switzerland; 4 Swiss Tropical and Public Health Institute, Basel, Switzerland; 5 University of Basel, Basel, Switzerland; 6 Division of Infectious Diseases & Hospital Epidemiology, University Hospital Basel, Basel, Switzerland; 7 ISGlobal, Barcelona Ctr. Int. Health Res, (CRESIB), Hospital Clínic - Universitat de Barcelona, Barcelona, Spain; Ghent University, BELGIUM

## Abstract

**Introduction:**

Scarce data are available on the epidemiology of hypertension among HIV patients in rural sub-Saharan Africa. We explored the prevalence, incidence and risk factors for incident hypertension among patients who were enrolled in a rural HIV cohort in Tanzania.

**Methods:**

Prospective longitudinal study including HIV patients enrolled in the Kilombero and Ulanga Antiretroviral Cohort between 2013 and 2015. Non-ART naïve subjects at baseline and pregnant women during follow-up were excluded from the analysis. Incident hypertension was defined as systolic blood pressure ≥ 140 mmHg and/or diastolic blood pressure ≥ 90 mmHg on two consecutive visits. Cox proportional hazards models were used to assess the association of baseline characteristics and incident hypertension.

**Results:**

Among 955 ART-naïve, eligible subjects, 111 (11.6%) were hypertensive at recruitment. Ten women were excluded due to pregnancy. The remaining 834 individuals contributed 7967 person-months to follow-up (median 231 days, IQR 119–421) and 80 (9.6%) of them developed hypertension during a median follow-up of 144 days from time of enrolment into the cohort [incidence rate 120.0 cases/1000 person-years, 95% confidence interval (CI) 97.2–150.0]. ART was started in 630 (75.5%) patients, with a median follow-up on ART of 7 months (IQR 4–14). Cox regression models identified age [adjusted hazard ratio (aHR) 1.34 per 10 years increase, 95% CI 1.07–1.68, p = 0.010], body mass index (aHR per 5 kg/m^2^ 1.45, 95% CI 1.07–1.99, p = 0.018) and estimated glomerular filtration rate (aHR < 60 versus ≥ 60 ml/min/1.73 m^2^ 3.79, 95% CI 1.60–8.99, p = 0.003) as independent risk factors for hypertension development.

**Conclusions:**

The prevalence and incidence of hypertension were high in our cohort. Traditional cardiovascular risk factors predicted incident hypertension, but no association was observed with immunological or ART status. These data support the implementation of routine hypertension screening and integrated management into HIV programmes in rural sub-Saharan Africa.

## Introduction

The use of antiretroviral therapy (ART) has transformed HIV disease from a rapidly lethal illness into a long-term chronic condition. With ART coverage reaching 15.8 million globally and around 12 million patients in sub-Saharan Africa by December 2015 [1], survival for people living with HIV has dramatically increased [2–4]. As survival rates improve, treatment focus is gradually being shifted towards the long-term management of HIV infection, comorbidities and ART-associated chronic complications. Among these emerging challenges, cardiovascular diseases occupy a prominent position as major sources of morbidity and mortality in HIV-infected patients [5, 6].

Rapid changes in lifestyle, together with a hypertension-prone genetic background in black Africans, are leading to the unfolding of a hypertension epidemic of presumably distinct characteristics from those observed outside the continent [7, 8]. Indeed, age-adjusted prevalences of hypertension in the region have already reached the highest levels in the world with an estimated prevalence of 30% [9, 10]. While there is consensus in the field about the increased risk of cardiovascular events among HIV patients [11–13], the relation of hypertension with HIV infection and ART remains controversial. Indeed, inconsistent and often contradictory reports of associations between HIV infection, ART exposure, specific antiretroviral drug regimens and HIV-related factors have been successively reported in the literature [14–22]. Interestingly, some widely used antiretroviral drugs have been linked to renal and cardiovascular adverse effects (i.e. tenofovir disoproxil fumarate [TDF] and nephrotoxicity; abacavir [ABC] and myocardial infarction), but their relevance in the hypertension scenario is not well defined [23–24]. This degree of uncertainty on the burden and epidemiology of hypertension is even higher for sub-Saharan African HIV populations, where only a limited number of studies have approached the subject from a mostly cross-sectional perspective [25–30].

Thus, unravelling the epidemiology of hypertension among HIV patients in sub-Saharan Africa stands out as an urgent task, and could potentially set the ground for a more optimal clinical management. In this context, our study aimed to explore the prevalence, incidence and risk factors of hypertension development among ART-naive HIV-infected patients enrolled in the Kilombero and Ulanga Antiretroviral Cohort (KIULARCO) in rural southern Tanzania.

## Methods

### Study design and setting

This is a longitudinal study based on prospectively collected data from participants enrolled in KIULARCO, an open, ongoing cohort that recruits HIV-infected individuals receiving care at the Chronic Diseases Clinic of Ifakara within the Saint Francis Referral Hospital (SFRH). The SFRH is the major healthcare facility in the Kilombero and Ulanga districts of the Morogoro region in southern Tanzania, serving an estimated target population of approximately 600.000 inhabitants. Since its establishment in 2004, more than 8000 patients have been recruited into care, being the first rural clinic accredited to be a Care and Treatment Centre of the National AIDS Control Program in Tanzania back in 2004 [31, 32].

### Study population

HIV-infected patients ≥ 15 years-old enrolled in KIULARCO between January 1, 2013 and March 2, 2015 were eligible for inclusion in this study. Non-ART naïve subjects at baseline, patients on transit from other healthcare facilities and individuals with less than 2 registered blood pressure measurement sessions within 6 months since recruitment were excluded. Pregnant women during the study period were not included in the analysis due to known effects of pregnancy on blood pressure and referral per protocol to a specialist clinic. Patient follow-up was closed on August 27, 2015.

### Definitions

#### Hypertension

Standardized blood pressure measurements using electronic monitors were performed by trained healthcare professionals. Measurements were obtained at routine clinical visits before ART initiation, 2 weeks and 3 months after ART initiation, and every 6 months thereafter. Hypertension status was based on the Eighth Report of the Joint National Committee on the Prevention, Detection, Evaluation and Treatment of High Blood Pressure (JNC-8) criteria [33]. Incident hypertension was defined as systolic blood pressure (SBP) ≥ 140 mmHg and/or diastolic blood pressure (DBP) ≥ 90 mmHg on 2 consecutive visits. Subjects with recorded previous history of hypertension and those with SBP ≥ 140 mmHg and/or DBP ≥ 90 mmHg on the first 2 blood pressure measurement sessions within 6 months since registration were classified as hypertensive at baseline and subsequently excluded from the longitudinal analyses.

#### Renal function

Renal function was assessed by the estimated glomerular filtration rate (eGFR), which was calculated from serum creatinine with the CKD-EPI formula. The resulting data were categorized following the chronic kidney disease criteria (eGFR < 60 ml/min/1.73 m^2^) from the KDIGO 2012 Clinical Practice Guideline for the Evaluation and Management of Chronic Kidney Disease [34].

#### Immunological status

CD4 counts were measured by flow cytometry (BD FACSCalibur, Franklin Lakes, NJ) after whole blood CD4, CD3, CD8 and CD45 staining with generic labelled antibodies. CD4 counts were categorized in the descriptive analysis according to clinically relevant cut-offs, following the Centers for Disease Control and Prevention (CDC) definition of AIDS (CD4 count < 200 cells/mm^3^) and the conventional threshold for very severe immunosuppression (CD4 count < 50 cells/mm^3^) [35].

#### Overweight and obesity

Nutritional status was estimated by the body mass index (BMI), calculated as weight (kg)/height^2^ (m^2^). Underweight, overweight and obesity were defined according to the WHO criteria (underweight if BMI < 18.5 kg/m^2^, overweight if 25 kg/m^2^ < BMI < 30 kg/m^2^, and obesity if BMI ≥ 30 kg/m^2^) [36].

### Ascertainment of covariates

Demographic and clinical data were anonymized and extracted from the KIULARCO electronic databases. All patients underwent a detailed and systematic clinical assessment done by a physician at baseline. Values for relevant variables were collected at baseline only and were not time-updated.

### Statistical analyses

Univariate and multivariate Cox proportional hazards models were fitted to assess the association of the time to develop hypertension with demographic, clinical and treatment covariates. The assumption of proportional hazards was verified by the Schoenfeld’s global test (p = 0.11). Patients were censored at time of loss to follow-up (LTFU), transfer to other healthcare centres, death, or end of the study. Kaplan-Meier survival estimates according to the independent risk factors at baseline were constructed and compared using the log-rank test. All data management and statistical analyses were conducted in Stata 13 (Stata Corp, College Station, TX).

### Ethics statement

Written informed consent was sought from all study participants at enrolment in KIULARCO at the Chronic Diseases Clinic of Ifakara after written and oral information on clinical data collection was provided. Consent was obtained from the parents or guardians of the minors included in KIULARCO. Ethical approval was granted by the local Ethical Committee of the Ifakara Health Institute, the Medical Research Coordination Committee of the National Institute of Medical Research of Tanzania, the Tanzanian Commission of Science and Technology, and the Ethics Committee of the University and State of Basel, Switzerland.

## Results

### Characteristics of the study population

**Fig 1:**
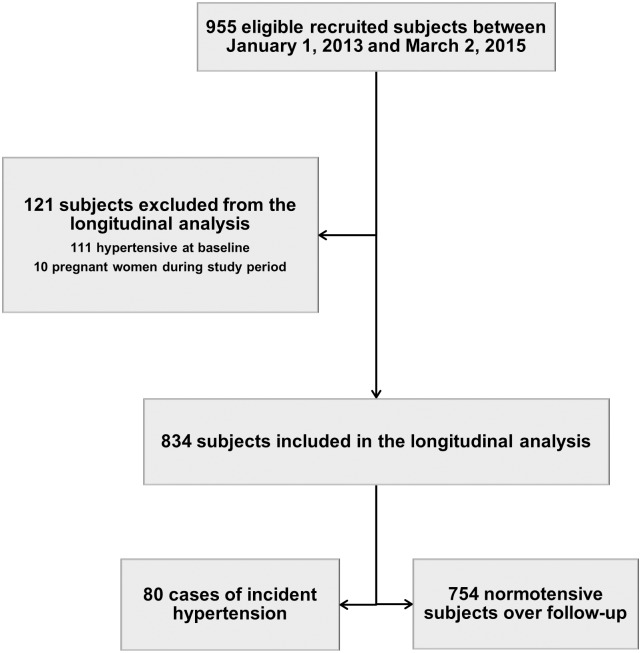
Patient flow chart.

Among 955 potentially eligible ART-naïve HIV-infected subjects, 111 (11.6%) were categorized as hypertensive at baseline and 10 women became pregnant during the study period. Thus, a total number of 834 ART-naïve individuals fulfilled the predefined inclusion criteria for the longitudinal study (Fig 1). Median age at recruitment was 38.2 years (IQR 32–46), 61.6% of participants were female and all were of African ethnicity. Median baseline CD4 count was 188 cells/μL (IQR 66–367) and 50.2% of patients had WHO III-IV stage. Underweight (Body Mass Index < 18.5 kg/m^2^) at enrolment was reported in 27.9% of individuals, whereas overweight or obesity (BMI ≥ 25 kg/m^2^) was seen in 11.8% of patients. A low basal eGFR (< 60 ml/min/1.73 m^2^) was present in 3.9% of participants. ART was started in 630 (75.5%) patients, with a median time of 15 days (IQR 7–39) from recruitment to treatment initiation and a median follow-up on ART of 7 months (IQR 4–14) at the time of analysis. ART-initiation criteria were defined as CD4 ≤ 350 cells/mm^3^ and/or WHO III/IV stage. The single-tablet, co-formulated combination of tenofovir disoproxil fumarate (TDF), lamivudine (3TC) and efavirenz (EFV) was the most common first-line ART regimen (55.7%), followed by the single tablet containing TDF, emtricitabine (FTC) and EFV (32.1%). Table 1 summarizes the baseline characteristics of the patients included in the cohort analysis according to hypertension status during follow-up. One hundred and eighty-two (21.8%) patients were lost to follow-up (LTFU), 56 (6.7%) were transferred to other centres and 47 died without having developed hypertension. Median times to LTFU or transfer were 185 days (IQR 103–269) and 244 days (IQR 161–297), respectively. LTFU was more frequent among patients not started on ART (41.7%) than among patients receiving ART (15.4%). A large majority of patients who opted for continuation of care at other clinics were female (80.4%), and the proportion of subjects who were not started on ART was higher among LTFU (46.7%) and transferred-out (30.4%) individuals when compared to those kept under follow-up (17.1%). Apart from these differences, the overall distributions of covariates across subjects in these groups were largely comparable. Characteristics of patients LTFU or transferred to other clinics before the onset of hypertension, as well as of those remaining in the cohort, are shown in Table 2. At the end of the study period, 534 (64.0%) individuals remained in active follow-up, 187 (22.4%) were LTFU, 62 (7.4%) had been transferred to other healthcare centres, and 51 (6.1%) had died.

**Table 1:** Summary of patient baseline characteristics according to the development of hypertension.

	Total Cohort	Hypertensive during follow-up	Normotensive during follow-up
	n = 834	n = 80	n = 754
**Age** (years)			
Median (IQR)	38.2 (32.3–45.6)	40.7 (34.7–50.9)	37.9 (32.1–45.4)
15–30 years, n (%)	135 (16.2)	10 (12.5)	160 (21.2)
31–40 years, n (%)	339 (40.6)	28 (35.0)	276 (36.6)
> 40 years, n (%)	360 (43.2)	42 (52.5)	318 (42.2)
**Gender**			
Male, n (%)	320 (38.4)	34 (42.5)	286 (37.9)
Female, n (%)	514 (61.6)	46 (57.5)	468 (62.1)
**BMI** (kg/m^2^)			
Median (IQR)	20.1 (18.3–22.3)	20.9 (18.5–24.1)	20.1 (18.2–22.2)
<18.5, n (%)	231 (27.9)	19 (24.0)	212 (28.4)
18.5–24.9, n (%)	498 (60.3)	44 (55.7)	454 (60.8)
25–29.9, n (%)	79 (9.6)	12 (15.2)	67 (8.9)
≥ 30, n (%)	18 (2.2)	4 (5.1)	14 (1.9)
**eGFR** (ml/min/1.73m^2^)			
Median (IQR)	127.8 (114.7–137.2)	123.9 (100.9–138.9)	127.9 (115.3–137.1)
≥ 60, n (%)	777 (96.1)	70 (90.9)	707 (96.6)
< 60, n (%)	32 (3.9)	7 (9.1)	25 (3.4)
**African ethnicity**, n (%)	834 (100)	80 (100)	754 (100)
**Smoking status**			
Non-smoker, n (%)	759 (93.2)	75 (94.9)	684 (93.1)
Current smoker, n (%)	55 (6.8)	4 (5.1)	51 (6.9)
**Alcohol consumption**			
No, n (%)	670 (82.3)	70 (88.6)	600 (81.6)
Yes, n (%)	144 (17.7)	9 (11.4)	135 (18.4)
**Tuberculosis comorbidity**			
No, n (%)	723 (86.7)	72 (90)	651 (86.3)
Yes, n (%)	111 (13.3)	8 (10)	103 (13.7)
**CD4 count** (cells/mm^3^)			
Median (IQR)	187.5 (66–367)	190 (73–330)	187 (64–374)
> 200, n (%)	369 (47.4)	35 (49.3)	334 (47.2)
50–200, n (%)	258 (33.2)	25 (35.2)	233 (33.0)
< 50, n (%)	151 (19.4)	11 (15.5)	140 (19.8)
**WHO stage** (I-IV)			
I-II, n (%)	398 (49.8)	43 (55.9)	355 (49.2)
III-IV, n (%)	401 (50.2)	34 (44.1)	367 (50.8)
**ART exposure during follow-up**			
ART-naive, n (%)	204 (24.5)	12 (15.0)	192 (25.5)
ART-exposed, n (%)	630 (75.5)	68 (85.0)	562 (74.5)
**ART initiation regimen**			
AZT+3TC+NVP, n (%)	9 (1.5)	3 (4.5)	6 (1.1)
AZT+3TC+EFV, n (%)	56 (9.1)	4 (6.0)	52 (9.5)
TDF+FTC+EFV, n (%)	197 (32.1)	24 (35.8)	173 (31.6)
TDF+3TC+EFV, n (%)	342 (55.7)	34 (50.7)	308 (56.3)
ABC+3TC+EFV, n (%)	6 (1.0)	2 (3.0)	4 (0.7)
TDF+FTC+LPV/r, n (%)	2 (0.3)	0	2 (0.4)
TDF+FTC+ATV/r, n (%)	2 (0.3)	0	2 (0.4)

BMI: body mass index; eGFR: estimated glomerular filtration rate; n: number of patients; ART: antiretroviral therapy; AZT: zidovudine; 3TC: lamivudine; NVP: nevirapine; EFV: efavirenz; TDF: tenofovir disoproxil fumarate; FTC: emtricitabine; ABC: abacavir; LPV/r: lopinavir/ritonavir; ATV/r: atazanavir/ritonavir. BMI: 8 missing values; eGFR: 25 missing values; smoking status: 20 missing values; alcohol consumption: 20 missing values; CD4 count: 56 missing values; WHO stage: 35 missing values; ART initiation regimen: 16 missing values;

**Table 2:** Summary of patient baseline characteristics according to patient follow-up status.

	LTFU	Transferred-out	Non-LTFU/transferred-out
	n = 182	n = 56	n = 596
**Age** (years)			
Median (IQR)	37.3 (31.7–43.8)	37.0 (32.7–41.9)	38.8 (32.5–46.3)
15–30 years, n (%)	43 (23.6)	6 (10.7)	94 (15.8)
31–40 years, n (%)	70 (38.5)	32 (57.1)	229 (38.4)
> 40 years, n (%)	69 (37.9)	18 (32.1)	273 (45.8)
**Gender**			
Male, n (%)	84 (46.2)	11 (19.6)	225 (37.8)
Female, n (%)	98 (53.8)	45 (80.4)	371 (62.2)
**Body mass index** (kg/m^2^)			
Median (IQR)	19.9 (18.3–21.8)	18.7 (17.5–20.5)	20.3 (18.3–22.7)
<18.5, n (%)	50 (27.8)	25 (44.7)	156 (26.4)
18.5–24.9, n (%)	116 (64.4)	27 (48.2)	355 (60.2)
25–29.9, n (%)	10 (5.6)	4 (7.1)	65 (11)
≥ 30, n (%)	4 (2.2)	0	14 (2.4)
**eGFR** (ml/min/1.73 m^2^)			
Median (IQR)	128.4 (117.4–137.6)	128.4 (116.3–139.5)	127.2 (113.0–138.1)
≥ 60, n (%)	163 (96.4)	53 (96.4)	561 (95.9)
< 60, n (%)	6 (3.6)	2 (3.6)	24 (4.1)
**African ethnicity,** n (%)	182 (100)	56 (100)	596 (100)
**Smoking status**			
Non-smoker, n (%)	158 (89.8)	54 (96.4)	547 (94.0)
Current smoker, n (%)	18 (10.2)	2 (3.6)	35 (6.0)
**Alcohol consumption**			
No, n (%)	140 (79.5)	47 (83.9)	483 (83.0)
Yes, n (%)	36 (20.5)	9 (16.1)	99 (17.0)
**Tuberculosis comorbidity**			
No, n (%)	156 (85.7)	46 (82.1)	521 (87.4)
Yes, n (%)	26 (14.3)	10 (17.9)	75 (12.6)
**CD4 count** (cells/mm^3^)			
Median (IQR)	229 (44–438)	137 (57–306)	184 (71–343)
> 200, n (%)	89 (53.0)	21 (41.2)	259 (46.3)
50–200, n (%)	36 (21.4)	18 (35.3)	204 (36.5)
< 50, n (%)	43 (25.6)	12 (23.5)	96 (17.2)
**WHO stage** (I-IV)			
I-II, n (%)	75 (42.6)	23 (41.8)	300 (52.8)
III-IV, n (%)	101 (57.4)	32 (58.2)	268 (47.2)
**ART exposure during follow-up**			
ART-naive, n (%)	85 (46.7)	17 (30.4)	102 (17.1)
ART-exposed, n (%)	97 (53.3)	39 (69.6)	494 (82.9)
**ART initiation regimen**			
AZT+3TC+NVP, n (%)	1 (1.1)	1 (2.6)	7 (1.5)
AZT+3TC+EFV, n (%)	9 (9.5)	3 (7.9)	44 (9.1)
TDF+FTC+EFV, n (%)	34 (35.7)	13 (34.2)	150 (31.2)
TDF+3TC+EFV, n (%)	46 (48.4)	21 (55.3)	275 (57.2)
ABC+3TC+EFV, n (%)	2 (2.1)	0	4 (0.8)
TDF+FTC+LPV/r, n (%)	1 (1.1)	0	1 (0.2)
TDF+FTC+ATV/r, n (%)	2 (2.1)	0	0

BMI: body mass index; eGFR: estimated glomerular filtration rate; n: number of patients; ART: antiretroviral therapy; AZT: zidovudine; 3TC: lamivudine; NVP: nevirapine; EFV: efavirenz; TDF: tenofovir disoproxil fumarate; FTC: emtricitabine; ABC: abacavir; LPV/r: lopinavir/ritonavir; ATV/r: atazanavir/ritonavir. BMI: 8 missing values; eGFR: 25 missing values; smoking status: 20 missing values; alcohol consumption: 20 missing values; CD4 count: 56 missing values; WHO stage: 35 missing values; ART initiation regimen: 16 missing values;

### Baseline prevalence and incidence of hypertension

One hundred and eleven subjects met the definition of hypertension at baseline, corresponding to a prevalence of 11.6% (95% confidence interval [CI] 9.7–13.8). Ten women who became pregnant during follow-up were subsequently excluded from the incidence calculation. The remaining 834 individuals contributed 7967 person-months (p-m) during the study period, with a median time of 231 days (IQR 119–421) of follow-up and a median number of clinical visits of 7 (IQR 4–11). Of these, 80 (9.6%) developed hypertension during follow-up (incidence rate 120.0 cases/1000 person-years [p-y] [95% CI 97.2–150.0]) after a median time of 144 days (IQR 56–289) after enrolment.

### Risk factors associated with development of hypertension

**Fig 2:**
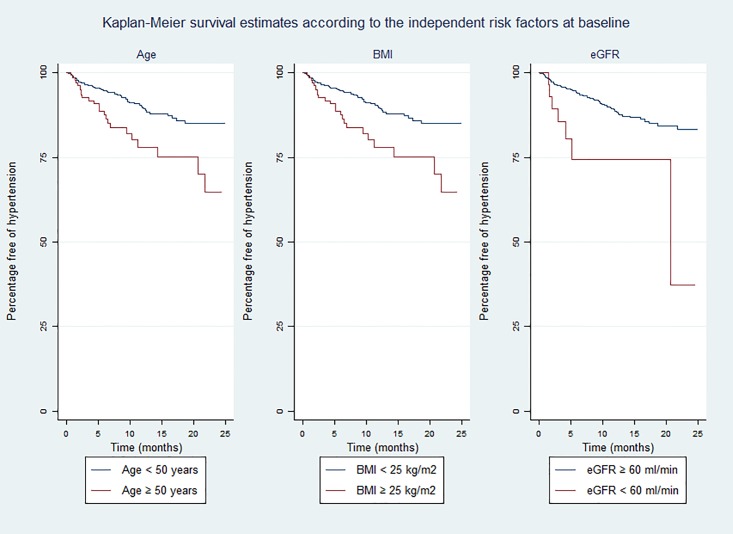
Kaplan-Meier survival estimates of hypertension development according to the independent risk factors at baseline: age (left), body mass index (centre) and estimated glomerular filtration rate (right). BMI: body mass index; eGFR: estimated glomerular filtration rate.

Age (adjusted hazard ratio [aHR] per 10 years: 1.34, 95% CI 1.07–1.68, p = 0.010), BMI (aHR per 5 kg/m^2^: 1.45, 95% CI 1.07–1.99, p = 0.018) and eGFR (aHR < 60 versus ≥ 60 ml/min/1.73 m^2^: 3.79, 95% CI 1.60–8.99, p = 0.003) were identified as independent risk factors of incident hypertension in our adjusted Cox regression model (Table 3). In addition, a trend was observed towards and association of female sex (aHR: 0.63, 95% CI 0.37–1.09, p = 0.097) and alcohol consumption (aHR: 0.50, 95% CI 0.23–1.12, p = 0.091) with lower incidence of hypertension. Kaplan—Meier estimates for the probability of remaining free of hypertension by the three independent risk factors are displayed in Fig 2. The survival curves show a higher proportion of observed hypertension events during the study period among those subjects with baseline eGFR < 60 ml/min/1.73 m^2^ (21.9% versus 9.01% for eGFR ≥ 60 ml/min/1.73 m^2^; log rank p = 0.0004), age at recruitment ≥ 50 years (28% versus 3.4% for age < 50 years; log rank p = 0.0006) and BMI ≥ 25 kg/m^2^ (16.5% versus 8.6% for BMI < 25 kg/m^2^; log rank p = 0.0671). Neither baseline CD4 count (aHR per 25 cell/mm^3^ increase: 0.98, 95% CI 0.94–1.02, p = 0.300) nor exposure to ART (aHR: 0.95, 95% CI 0.42–2.13, p = 0.907) were associated with the development of hypertension in our cohort.

**Table 3:** Cox regression analysis of risk factors for hypertension development.

	Univariate model		Multivariate model	
Variable	Unadjusted HR (95% CI)	*p* value	Adjusted HR (95% CI)	*p* value
**Age (per 10-year increase)**	1.33 (1.10–1.60)	0.004	1.34 (1.07–1.68)	0.010
**Female gender**	0.74 (0.47–1.15)	0.180	0.63 (0.37–1.09)	0.097
**BMI (per 5 kg/m^2^ increase)**	1.32 (1.02–1.71)	0.037	1.45 (1.07–1.99)	0.018
**eGFR (< 60 ml/min/1.73 m^2)^**	3.74 (1.71–8.18)	0.001	3.79 (1.60–8.99)	0.003
**Smoking**	0.81 (0.30–2.22)	0.682	0.61 (0.19–2.04)	0.427
**Alcohol consumption**	0.57 (0.28–1.14)	0.112	0.50 (0.23–1.12)	0.091
**CD4 count (per 25 cell/mm^3^ increase)**	0.98 (0.95–1.01)	0.194	0.98 (0.94–1.02)	0.300
**ART exposure**	1.04 (0.56–1.93)	0.906	0.95 (0.42–2.13)	0.907

BMI: body mass index; eGFR: estimated glomerular filtration rate; ART: antiretroviral therapy.

## Discussion

This prospective longitudinal study is among the few to assess the incidence of hypertension in a broadly inclusive population of HIV-positive subjects recruited into care in a sub-Saharan African setting. Key findings were the 12% prevalence of hypertension at recruitment, the 10% incidence of hypertension during a relatively short follow-up, the association of well-known cardiovascular risk factors with incident hypertension, and the lack of association of incident hypertension with baseline CD4 counts or ART status.

As ART programmes are globally scaled-up and AIDS-related deaths are reduced across developing countries, the management of non-communicable comorbidities such as hypertension is emerging as an essential part of HIV care in resource-limited settings. Yet, longitudinal data on incident hypertension in HIV patients in sub-Saharan Africa are largely lacking. In the present study, we found both a high prevalence at recruitment (11.6%) and a high incidence of hypertension among patients initiating HIV care in a rural healthcare facility in southern Tanzania. Eighty patients (9.6%) developed hypertension over a relatively short period of cohort follow-up (median time of 231 days), leading to an estimated incidence rate of 120.0 cases/1000 p-y (95% CI 97.2–150.0). These results are comparable to those recently reported in a similar study restricted to patients on ART in rural Uganda (111.5 cases/1000 p-y, 95% CI 101.9–121.7) [30]. Remarkably, these incidences are more than 1.5-fold higher than that found in the D:A:D cohort (72.1 cases/1000 p-y, [95% CI 68.2–76.0]) [17], a multinational study comprising mostly Caucasian patients from resource-rich settings in the United States, Europe and Australia, suggesting a higher incidence of hypertension among people living with HIV in rural sub-Saharan Africa.

Well-known cardiovascular risk factors such as age, BMI and eGFR were confirmed as strong risk factors of hypertension. The impact of HIV- or ART-specific hypertension risk factors in the HIV patient population remains a controversial issue, with conflicting results reported in different settings [14–22]. While having been linked by several studies to an increased risk of hypertension, ART exposure has been also associated with decreased odds of hypertension in a large South African population-based survey. Yet, lack of adjustment for immunological status or disease stage, very restrictive ART initiation criteria and short duration of treatment may underlie these findings [27]. In our cohort, we did not find any association between hypertension and baseline CD4 count or ART exposure. It should be noted that ART exposure was also short in our study, and that most patients initiated ART during follow-up, which may have limited our power to detect statistical differences between ART-exposed and unexposed subjects. Moreover, this imbalance between groups was exacerbated by a more extensive LTFU among individuals not started on ART. A great majority of the patients initiated ART regimens based on the single tablet triple combination of TDF / (3TC or FTC) / EFV, with only marginal numbers exposed to other antiretroviral drugs. This distribution of ART regimens in our cohort precluded a comprehensive analysis of whether exposure to specific antiretroviral drugs was associated with an increased risk of hypertension. Noteworthy, a recent analysis from the D:A:D study failed to find any clinically relevant independent association between exposure to individual ART drugs and hypertension [37]. ART effects on body mass or immune reconstitution have been proposed as potential drivers of increased hypertension risk among HIV patients [28, 30]. Also, exposure to TDF has been associated with accelerated eGFR decrease in the first 6–12 months after ART initiation among African HIV patients with no or mild renal dysfunction at baseline [38]. Although the reported overall effects were modest, the possible impact of secondary nephrotoxicity on hypertension rates deserves consideration given the large proportion of patients receiving TDF-based ART in our cohort. However, we were not able to investigate these factors due to our study design without time-updated variables and a short follow-up time. Extended cohort follow-up will be required in order to assess hypertension risk factors and temporal trends in the longer run. Overall, our results are consistent with those of prior studies showing a remarkably elevated risk of hypertension among HIV patients and individuals of African ancestry and support universal hypertension screening strategies regardless of baseline CD4 count or ART exposure.

This study has some limitations. The lack of an HIV-negative control group hinders our ability to examine the impact of HIV infection on hypertension. It therefore remains uncertain whether and to which degree the high observed incidence in our cohort may be reflecting population-wide disease trends. In this context, data from several studies have suggested that HIV infection could be associated with lower hypertension prevalence and decreased blood pressure with respect to uninfected individuals, an effect that appears to be only partially mediated by BMI changes [22, 25, 27]. Although measurement bias of hypertension events was minimised by the definition requirement of two consecutive abnormal BP values, the non-availability of data on concurrent antihypertensive drug treatment may have led to non-differential misclassification and underestimation of the incidence of hypertension. Antihypertensive treatment and adherence rates are expected to be low in our cohort due to severe financial barriers to drug use, but detailed data are lacking. Also, relevant variables such as serum lipid levels, HIV viral load, or use of traditional medications potentially contributing to hypertension were not routinely ascertained during clinical practice and could not be incorporated into our regression analyses. Lastly, LTFU and transfer to other clinics were frequent events during follow-up and may complicate the interpretation of our results. The hereby reported high patient drop-out rates could be explained in the context of restricted access to the hospital facilities during the rainy season due to impaired communications, and common continuation of HIV care in non-referral clinics, usually closer to the patients’ place of residence. Importantly, no major overall differences between the identified risk factors for hypertension at baseline of completers and dropout patients could be identified, suggesting a low potential for the introduction of significant bias into our analyses. Despite these drawbacks, our study provides significant new insights into the epidemiology of hypertension in rural sub-Saharan Africa and has important implications for public health programmes in the region.

Health systems in sub-Saharan Africa are expected to face an unprecedented epidemic of both communicable and emerging chronic diseases in the near future [39]. This double burden of disease will arguably represent a major strain for still under-resourced health services across the continent. Hypertension and other non-communicable diseases share a number of similarities with HIV, such as the chronic evolution of disease, the importance of behavioural changes and the need for regular follow-up and optimal treatment adherence, which make them attractive for an integrated management approach. Leveraging synergies between HIV and non-communicable diseases care without compromising the efficiency of HIV programmes is one of the challenges lying ahead. This study has shown that the implementation of routine hypertension screening represents a feasible strategy for hypertension diagnosis within an HIV rural programme. Likewise, further research that evaluates the outcomes of interventions promoting hypertension treatment and control among HIV patients recruited into KIULARCO is warranted. Encouragingly, the integration of HIV, hypertension and diabetes management at chronic diseases clinics in Cambodia has already resulted in efficiency gains for the services, good patient acceptance and favourable HIV clinical outcomes [40]. Expanded access to antihypertensive drugs will be essential, however, if similar integrated HIV programmes are to be successful in sub-Saharan African settings.

## Conclusions

In summary, our study shows that both baseline presence and early development of hypertension after recruitment into care are common among HIV patients in rural Tanzania. Well-known risk factors were associated with incident hypertension, but no association was observed with baseline immunological status or ART exposure. These data support the implementation of universal routine hypertension screening for HIV patients and highlight the need for the incorporation of evidence-based non-communicable diseases management strategies into integrated HIV programmes in rural sub-Saharan Africa.

## Supporting information

S1 File Minimal dataset. (XLS)
